# Spray‐Dried Wheat Gluten Protein Hydrolysate Microcapsules: Physicochemical Properties, Retention of Antioxidant Capability, and Release Behavior Under Simulated Gastrointestinal Digestion Conditions

**DOI:** 10.1002/fsn3.4662

**Published:** 2024-12-19

**Authors:** Benyamin GanjiVtan, Seyyed Hossein Hosseini Ghaboos, Alireza Sadeghi Mahoonak, Taher Shahi, Neda Farzin

**Affiliations:** ^1^ Department of Food Science and Engineering Azadshahr Branch, Islamic Azad University Azadshahr Iran; ^2^ Food Science and Technology Research Center of East Golestan, Azadshahr Branch Islamic Azad University Azadshahr Iran; ^3^ Faculty of Food Science & Technology Gorgan University of Agricultural Sciences and Natural Resources Gorgan Iran; ^4^ Faculty of Agricultural and Natural Resources, Azadshahr Branch Islamic Azad University Azadshahr Iran; ^5^ Department of Animal Science, Azadshahr Branch Islamic Azad University Azadshahr Iran

**Keywords:** bioactive peptides, enzymatic hydrolysis, gluten, potato starch, spray drying

## Abstract

Wheat gluten is a by‐product of the wheat starch industry, rich in bioactive peptides. Spray drying is an effective method for improving the stability of bioactive compounds. So, the aim of this study was to produce gluten hydrolysate by different proteases (alcalase, pancreatin, and trypsin) at different times (40–200 min). The hydrolysate with the strongest antioxidant potential (produced by pancreatin after 200 min of hydrolysis) was encapsulated by spray drying. The effect of wall material's type (maltodextrin, potato starch, and their combination at different ratios) on the encapsulation efficiency, physicochemical properties (moisture content, solubility, water activity, tapped and bulk density, and hygroscopicity), release behavior under simulated gastrointestinal digestion conditions, and morphology of microcapsules were evaluated. The microcapsules produced by maltodextrin and potato starch at a 30:70 ratio possessed the highest water activity (0.36), encapsulation efficiency (85.79%), and moisture content (8.2%). An increase in maltodextrin concentration increased the solubility, bulk, and tapped density. SEM images showed that microparticles were spherical with wrinkled surfaces. The microcapsules showed higher stability than free gluten hydrolysate. The combination of maltodextrin and potato starch at a 30:70 ratio could control the release of gluten hydrolysate under simulated gastrointestinal conditions. As a result, the use of maltodextrin and potato starch carriers at a 30:70 ratio in spray drying could effectively protect the bioactive properties of gluten hydrolysate and control its release.

## Graphical abstract

This study aimed to the effect of hydrolysis time and enzyme type on the antioxidant activity of gluten hydrolysate. Then the sample with the strongest antioxidant potential was encapsulated by spray drying using different wall materials. The physicochemical properties, retention of antioxidant properties, and release behavior under simulated gastrointestinal conditions were investigated.

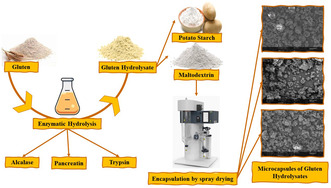

## 1 Introduction

Recently, there has been an increasing demand for functional food products with the least synthetic additives (Rezazadeh‐Bari et al. 2019), which has drawn the attention of researchers to nutritious and healthy products of this type (Carneiro et al. 2013; Halim et al. 2018; Kaveh, Mahoonak, and Sarabandi 2020; Murthy et al. 2017; Wang et al. 2020). Bioactive peptides and protein hydrolysates offer various bioactive potentials, including anticancer, antimicrobial, antioxidant, hypocholesterolemic, and antihypertensive effects (Jemil et al. 2016; Kaprasob et al. 2022; Kaveh et al. 2019a; Kumar, Muthu Kumar, and Tiku 2021; Umayaparvathi, Meenakshi, et al. 2014). These health‐beneficial properties have increased the researcher's attention to produce bioactive peptides and protein hydrolysate from various sources such as fenugreek seeds (Kaveh et al. 2022), pumpkin seeds (Mahoonak and Kaveh 2022), mung bean (Liu et al. 2022), Turkmen melon seed (Alvand et al. 2022), skipjack fish processing wastes (Kaveh et al. 2023), sheep visceral (Meshginfar et al. 2014), and oyster (Ma et al. 2022). In this regard, one of the highly potent sources is wheat gluten, a plant protein with many applications in food and non‐food products. Wheat gluten makes up a large proportion of the total wheat protein (about 85%) and has a unique amino acid composition consisting of glutenin and gliadin (Nongonierma et al. 2017; Suetsuna and Chen 2003). Wheat gluten is a by‐product of the wheat starch industry, which is commercially available at a relatively reasonable cost in large amounts (Cian, Vioque, and Drago 2015). Although bioactive peptides have several health benefits, their applications in the food and pharmaceutical industries have been significantly limited due to issues such as poor solubility in lipids, low bioavailability, uncontrolled delivery, and physicochemical instability in undesirable conditions like high temperatures, light, and oxygen. Additionally, their bitter taste poses another challenge (Hesarinejad et al. 2021; Li, Paulson, and Gill 2015). In this context, a promising approach is to utilize an appropriate encapsulation system, defined as a technique for protecting a bioactive compound (core) through its entrapment using a substance known as an encapsulant (Drosou, Krokida, and Biliaderis 2017).

Spray drying is a favorable and common encapsulation method with various advantages, such as short time of processing, low thermal stresses, easy handling, and reasonable processing cost (Shishir and Chen 2017). This technique has been used for the encapsulation of various protein hydrolysates such as chicken meat protein hydrolysate (Kurozawa, Park, and Hubinger 2011), egg white (Chen, Chi, and Wei 2012), pink perch meat (Murthy et al. 2017), soybean (Wang et al. 2020), non‐penaeid shrimp (Dhanabalan et al. 2020), and bighead carp skin (Dong, Yan, and Zhang 2022).

It has been documented that some tensions may be applied to proteins and bioactive peptides through encapsulation by spray‐drying, such as increased temperature in protein contact with the drying air, shear stress during their atomization to small droplets, and the exposure to the interface of air‐liquid at the surface of droplet may lead to aggregation and denaturation of bioactive peptides and protein hydrolysate and consequently may have an adverse effect on their functional and health‐beneficial properties (Ajmera and Scherließ 2014; Lee 2002). Therefore, adequate protection of bioactive peptides and protein hydrolysate is critical during the process of spray‐drying. In this regard, the applied wall materials have an incredible role in protecting the bioactivity of encapsulating compounds. Among the suitable wall materials, MD is one of the most commonly used. MD has various advantages including high water solubility, low viscosity in high concentration, reasonable cost, acceptable aroma, and adequate protection against heat and oxidation (Fernandes et al. 2008; Kaveh et al. 2018). PS is another suitable wall material due to its inexpensive, easy availability, food‐grade ability, and biodegradability (Morán et al. 2021). To our knowledge, there is no study evaluating the effect of MD and PS on the physicochemical properties, antioxidant activity, and release of gluten protein hydrolysate. So, as the encapsulation by spray drying is a promising method to protect bioactive compounds such as gluten hydrolysate from undesirable conditions, and to control its release rate at simulated gastrointestinal conditions, the aims of this study were: (1) production of gluten hydrolysate as a high‐value product through enzymolysis using alcalase, pancreatin, and trypsin at various hydrolysis times (40–200 min), (2) evaluation of the antioxidant potential of wheat gluten hydrolysates, assessing DPPH radical scavenging activity, Fe^2+^ chelating activity, and total antioxidant capacity, (3) encapsulation of the hydrolysate with maximum antioxidant activity via spray drying, using different ratios of MD and PS as wall materials, (4) assessment of physical properties including encapsulation efficiency, moisture content, water activity, solubility, hygroscopicity, and density, as well as retention of antioxidant activity, microstructural properties analyzed by SEM, and release behavior.

## 2 Materials and Methods

Powdered gluten was obtained from the Khosheh Zarrin company, Bojnurd, Iran. Pancreatin from porcine pancreas, Alcalase 2.4 L. (Novo Nordisk, Bagsvaerd, Denmark), trypsin, pepsin, trichloroacetic acid (TCA), 1, 1‐diphenyl‐2‐picrylhydrazyl (DPPH^1^), and ferrozin were obtained from Merck company. Other used chemical compounds were purchased from Sigma‐Aldrich.

### 2.1 Production of Wheat Gluten Hydrolysate

The hydrolysis was performed at optimum conditions of pancreatin (40°C, pH 7.4), alcalase (50°C, pH 7.0), and trypsin (37°C, pH 8.0) based on the method of Kaveh et al. (2022). Briefly, wheat gluten (containing 90.42% protein) was dissolved in phosphate buffer (0.2 M) at 5% (*w*/*v*). After setting the temperature and pH of each sample to optimum levels, the enzymes were added to each solution at a concentration of 2% (*w*/*w*). Then, the hydrolysis process was done for 40, 80, 120, 160, and 200 min. For the inactivation of enzymes, the samples were heated (95°C, 5 min). Then, the centrifuging was done at 8000 ×*g* for 15 min. After that, the supernatants of each treatment were freeze‐dried and kept at −18°C till further experiments. The samples produced by pancreatin, trypsin, and alcalase were called GH‐Pan, GH‐Try, and GH‐Alc, respectively.

### 2.2 Degree of Hydrolysis (DH)

For the determination of DH, TCA (0.44 M) and each hydrolysate suspension were mixed at a ratio of 1:1 (*v*/*v*). Then incubation of the mixture was done at 4°C for 15 min. After that, the centrifuging was done at 8000 ×*g* for 15 min. In the next step, the soluble proteins in the supernatant were evaluated using the Bradford method (Bradford 1976). The DH was measured by equation (1).

(1) $$\begin{array}{cc} \mathrm{DH}\left(\%\right)= & \text{protein}\left(\mathrm{TCA}+\text{supernatant}\right)/\\ & \text{protein}\;\left(\text{hydrolysate suspension}\right)\;\times 100 \end{array}$$

### 2.3 Antioxidant Activity Assays of Wheat Gluten Hydrolysate (GH)

#### 2.3.1 DPPH Radical Scavenging Activity

Methanolic DPPH solution at a concentration of 0.2 mM was added to each hydrolysate solution (40 mg/mL) at 1:1 ratio. The solution was vortexed for 30 s and kept in the dark for 45 min (Kaveh et al. 2019b). After that, the absorbance of samples was measured at 517 nm and the DPPH radical scavenging activity was calculated by equation (2):

(2) $$\text{DPPH Scavenging activity}\left(\%\right)=\left[\left({\text{Absorbance}}_{\text{control}}-{\text{Absorbance}}_{\text{sample}}\right)/{\text{Absorbance}}_{\text{control}}\right]\times 100$$

#### 2.3.2 Fe^2+^ Chelating Activity

Briefly, 1.0 mL of each hydrolysate solution at a concentration of 40 (mg/mL) was mixed with 1.85 mL bidistilled water and vortexed for 30 s. In the next step, 0.05 mL of FeCl_2_ solution (2 Mm) and 0.1 mL of ferrozine solution at a concentration of 5 (mM) was added to the mixture and vortexed for 60 s. After that, the mixture was kept at 25°C for 15 min. Then, the absorbance was read at 562 nm and the Fe^2+^ chelating activity was measured by equation (3):

(3) $${\mathrm{Fe}}^{2+}\text{chelating activity}\left(\%\right)=\left[\left(A_{c}-A_{s}\right)/A_{c}\right]\times 100$$

#### 2.3.3 Total Antioxidant Activity

Firstly, each GH hydrolysate solution (40 mg/mL) was mixed with a reagent solution (sodium phosphate (28 mM), sulfuric acid (0.6 M), and ammonium molybdate (4 mM)) at a ratio of 1:10. After that, the incubation was done at 90°C for 90 min, and then samples were cooled to 25°C. Finally, the absorbance of samples was determined at 695 nm (Prieto, Pineda, and Aguilar 1999).

### 2.4 Microencapsulation of GH by Spray‐Drying

After analysis of the antioxidant activity of GHs produced by the mentioned enzymes, the GH produced by pancreatin activity after 200 min showed the highest antioxidant potential and was selected for encapsulation by spray‐drying. The carriers were MD and PS at 10% and 2% (*w*/*w*) concentrations, respectively. The proportion volume ratios of MD to PS were 100/0, 70/30, 50/50, 30/70, and 0/100. The GH was added to carrier solutions at a 1:2 ratio and was mixed at 1000 rpm for 30 min at 25°C. The produced GH microcapsules were called treat 1, 2, 3, 4, and 5, respectively. The process of spray drying was done by a laboratory spray dryer (DSD‐02, Dorsa Teb, Iran) with a nozzle with a diameter of 1.5 mm at a constant feed flow rate of 10 mL/min and inlet temperature of 120°C. The constant air compression pressure and the airflow were 40 m^3^/h and 6 bar, respectively. The obtained microcapsules were packed in sealed bags and stored at −8°C until the next experiments (Esquivel‐González et al. 2022).

### 2.5 Analysis of GH Microcapsules

#### 2.5.1 Encapsulation Efficiency (EE)

The GH microcapsules (100 mg) were mixed with 1 mL of potassium phosphate buffer (pH = 8 and 0.1 M). The mixture was vortexed for 15 min at 25°C. The surface protein content (SPC) and the total protein content (TPC) in the feed solution of the spray dryer before the process were measured by the Bradford method. EE (%) was calculated using the equation (4):

(4) $$\mathrm{EE}\;\left(\%\right)=\frac{\mathrm{TPC}\;\text{in feed solution}-\mathrm{SPC}\;\text{in encapsulated powder}}{\mathrm{TPC}\;\text{in feed solution}}\times 100$$

#### 2.5.2 Moisture Content and Water Activity (aw)

The moisture content of the GH microcapsules was calculated based on the AOAC (2012) procedure. To determine aw, the samples were analyzed by water activity meter (Labswift, Novasina, Switzerland).

#### 2.5.3 Solubility

Briefly, GH microcapsules were added to bidistilled water at 1% and mixed at 500 rpm for 5 min. The solution was then centrifuged at 4000 rpm for 5 min. The supernatant was dried (105°C for 5 h). The difference in weight between the dried solid and the initial powder was used to calculate the percentage of solubility (Kaveh et al. 2018).

#### 2.5.4 Hygroscopicity

Each sample (5 g) was placed in a desiccator containing a NaCl solution with a relative humidity of 75% and kept at 25°C for 7 days. The hygroscopicity of the samples was expressed as a gram of adsorbed moisture per 100 g of dry solids (Cai and Corke 2000).

#### 2.5.5 Density (Bulk and Tapped Density)

For the calculation of bulk density (g/ml), each sample (2 g) was gently added into a cylinder (10 mL). The occupied volume was recorded, and the ratio of mass to the occupied volume was reported as the value of bulk density. After that, about 400 beats were applied to the graduated cylinder, and tapped density was measured by stopping the volume changes of the sample (Jangam and Thorat 2010).

#### 2.5.6 Release Profile Under Simulated Digestion of Gastric Fluid (SGF) and Intestinal Fluid (SIF)

Briefly, the SGF medium contained pepsin (0.32%) and NaCl (0.2%) with a final pH of 2, and the SIF medium contained monobasic potassium phosphate (50 mM) and pancreatin (0.1%) with a final pH of 7. The GH microcapsules (500 mg) were placed in dialysis tubes, and these were placed in beakers containing SGF (500 mL). The beakers were incubated (37°C, 2 h) with constant stirring of 50 rpm. At 0, 30, 60, 90, and 120 min intervals, 0.1 mL of SGF was removed and replaced with fresh SGF medium. After 2 h, the dialysis tubes were transferred to a beaker containing SIF and incubated again (37°C, 4 h). At 0, 60, 120, 180, and 240 min intervals, 0.1 mL of SIF was removed and replaced with fresh SIF medium (Bao et al. 2023).

#### 2.5.7 Surface Morphology Analysis by Scanning Electron Microscopy (SEM)

A small quantity of each microcapsule was put on a double side adhesive tape fixed to SEM stubs and coated with a thin layer of gold. The images of each microcapsule were captured by scanning electron microscope (Zeiss EVO 40, Germany) (Tupuna et al. 2018).

#### 2.5.8 Stability of DPPH Radical Scavenging Activity of GH Microcapsules

The stability of GH microcapsules and free GH was performed by evaluating their DPPH radical scavenging activity stored in glass vials at 25°C for 30 days (da Silva et al. 2013).

### 2.6 Statistical Analysis

All the experiments were performed in triplicate, and the data were revealed as the means ± SD. The data was analyzed statistically based on the analysis of variance (ANOVA) followed by Duncan's test (*p* < 0.05) by the SPSS program.

## 3 Results and Discussion

### 3.1 Production of Wheat Gluten Hydrolysate (GH)

#### 3.1.1 Degree of Hydrolysis (DH)

**FIGURE 1:**
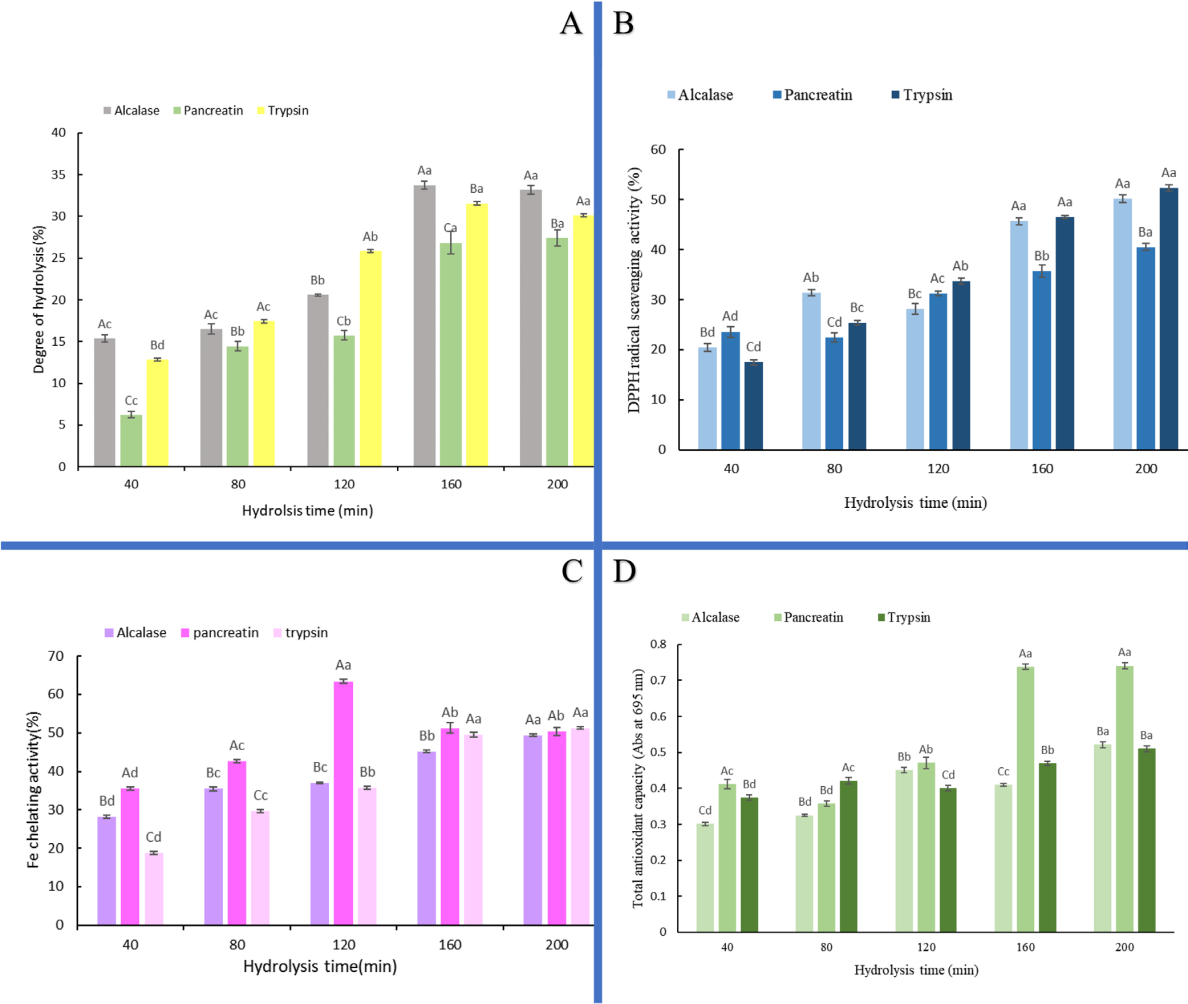
The effect of hydrolysis time and enzyme type on (A) degree of hydrolysis, (B) DPPH radical scavenging activity, (C) Fe^2+^ chelating activity, and (D) total antioxidant capacity of gluten hydrolysate. Different lowercase and capital letters indicate significant statistical differences at different hydrolysis times and at the same hydrolysis time, respectively (p < 0.05).

DH is a sign of the peptide bond's hydrolysis. Higher DH shows the production of peptides with shorter chain lengths (Farzanfar et al. 2024). The DH of hydrolysates was 6.22%–33.74%, depending on the type of applied enzymes and the time of hydrolysis (Figure 1A). The DH of all samples increased markedly by progressing the hydrolysis process from 40 to 200 min (*p* < 0.05). Increasing DH was revealed by Khantaphant, Benjakul, and Ghomi (2011) in the hydrolysis of brownstripe red snapper's muscle (Khantaphant, Benjakul, and Ghomi 2011). Also, Mahdavi‐Yekta, Nouri, and Azizi (2019) reported that increasing the hydrolysis time from 60 to 210 min resulted in higher DH of quinoa hydrolysate, as the DH increased from 7% to 14%. Also, Alahmad et al. (2023) revealed that higher DH was achieved by increasing hydrolysis time from 1 to 6 h in hydrolysis of Bighead Carp. After 200 min of hydrolysis, the DH of hydrolysates was in the order of alcalase > trypsin > pancreatin, which shows the higher activity of alcalase in cleavage peptide bonds compared to other used proteases (Kaveh et al. 2022). The differences in the obtained DH can be related to the enzyme's mechanisms of action, which can be endo‐ or exo‐peptidase (Kaveh et al. 2024). At first, the slope of DH increasing by all enzymes was high in the first 160 min and then decreased. This finding can result from inactivation of the applied proteases, reduction of available peptide bonds for hydrolysis, or the inhibition effect of produced peptides (Guérard, Guimas, and Binet 2002; Intarasirisawat et al. 2012). Similarly, Idowu et al. (2019) reported a reduction in the hydrolysis rate of salmon frames by papain and alcalase, and they revealed that alcalase produced hydrolysate with higher DH (Idowu et al. 2019).

### 3.2 Antioxidant Activity Assays of Wheat Gluten Hydrolysate (GH)

#### 3.2.1 DPPH Radical Scavenging Activity

DPPH• is a free radical that has a maximum absorbance at 517 nm (with a deep purple color). It can be scavenged by encountering a proton‐donating compound, which reduces absorbance at 517 nm (Akar, Küçük, and Doğan 2017). As depicted in Figure 1B, the DPPH radical scavenging capacity of hydrolysates was 17.48%–52.36%. At almost all hydrolysis times (except 40 min), samples produced by trypsin and alcalase showed stronger antioxidant activity than hydrolysates produced by pancreatin. For example, at the end of hydrolysis, the DPPH radical scavenging activity of samples was in the order of GHs produced by alcalase (50.18%) = trypsin (52.36%) > pancreatin (40.52%). There was no considerable difference between the antioxidant activity of hydrolysates produced by alcalase and trypsin at 200 min of hydrolysis (*p* > 0.05). Similarly, Bing et al. (2024) reported that trypsin hydrolysate showed the highest DPPH radical scavenging activity in comparison to other hydrolysates (Bing et al. 2024). The DPPH radical scavenging activity of all samples increased by progressing the hydrolysis, which can indicate an increase in hydrolysates' hydrogen‐donating activity. As mentioned in the previous section, alcalase and trypsin produced hydrolysates with higher DH than pancreatin. It has been reported that there is a direct correlation between the DPPH radical scavenging activity and DH, as peptides with higher DH are stronger DPPH radical scavengers. Following this finding, Khantaphant and Benjakul (2008) reported that the gelatin hydrolysate with higher DH showed stronger DPPH radical scavenging potential than hydrolysate with lower DH due to higher hydrogen‐donating activity (Khantaphant and Benjakul 2008). These are following the results of Lassoued et al. (2015) and de Castro et al. (2017) in hydrolysis of Thornback ray skin gelatin and white bean protein, respectively. Generally, it has been documented that the DPPH radical scavenging activity of protein hydrolysates depends on various factors including enzyme type, hydrolysis conditions (temperature, pH, and enzyme concentration), degree of hydrolysis, peptide sequences, the molecular distribution, and compositions of amino acid (Ahmed, Taie, and Abdel Wahab 2023; Sarmadi and Ismail 2010; Sarringkarin and Laokuldilok 2017). In this regard, Elavarasan and Shamasundar (2021) reported that the DPPH radical scavenging ability of freshwater carp protein hydrolysate depends on the amino acid composition and DH (Elavarasan and Shamasundar 2021).

#### 3.2.2 Fe^2+^ Chelating Activity

Metal ions, including Fe^2+^, are the accelerators of lipid oxidation and, consequently, the production of alkoxyl radicals, which can have undesirable effects on food quality (Mandel et al. 2006). The Fe^2+^ chelating activity of GHs is shown in Figure 1C. In hydrolysis with trypsin and alcalase, a considerable increase in the Fe^2+^ chelating activity of GHs was obtained by increasing the time of hydrolysis from 40 to 200 min (*p <* 0.05), but in hydrolysis with pancreatin, the increment in hydrolysis time from 40 to 120 min increased the chelating activity of GHs and more increase in hydrolysis time had a negative effect. Generally, the lowest (18.74%) and highest (63.41%) Fe^2+^ chelating activity was related to GH produced by trypsin at 40 min of hydrolysis and GH produced by pancreatin at 120 min of hydrolysis. The increase in Fe^2+^ chelating activity of hydrolysates by hydrolysis can result from the enhancement of carboxylic groups in the side chain of the basic and acidic amino acids (Zhang, Luo, and Wang 2011). Also, the increase of Fe^2+^ chelating ability of protein hydrolysate by progressing hydrolysis can be due to the increase in the number of ionic reactions and active sites by the release of free amino acids (Islam et al. 2021). After 200 min of hydrolysis, the Fe^2+^ chelating potential of all GHs produced by alcalase, pancreatin, and trypsin did not differ significantly (*p >* 0.05). It is documented that the chelation of metal ions such as Fe^2+^ and Cu^2+^ can limit oxidative stress by preventing the participation of the ions in the Fenton reaction (Torres‐Fuentes, Alaiz, and Vioque 2012). It has been reported that the ability of protein hydrolysates in chelating metal ions depends on different factors, including molecular weight, degree of hydrolysis, hydrolysis time, amino acid composition, and the enzyme's function in cleavage of peptide bonds (Carrasco‐Castilla et al. 2012; Kaveh et al. 2022). Moreover, it has been reported that the presence of histidine at the N‐terminal of bioactive peptides is effective in metal ion chelating due to the presence of the imidazole group (Ambigaipalan and Shahidi 2017).

These findings are in accordance with the results of Zheng et al. (2015), Zhang, Ding, and Li (2021), and El Hajj et al. (2023) in hydrolysis of corn glutenin, mung bean, soy and pea protein isolate, respectively (El Hajj et al. 2023; Zhang, Ding, and Li 2021; Zheng et al. 2015). Also, Islam et al. (2021) revealed that the increase in DH from 11.96% to 19.52% led to the increment of Fe chelating activity of grass turtle protein hydrolysate from 45% to 60% (Islam et al. 2021).

#### 3.2.3 Total Antioxidant Capacity

Phosphomolybdenum method is a quantitative assay that evaluates the antioxidant capacity of compounds. It is based on the reduction of molybdenum (VI) to green molybdenum (V) by antioxidant compounds at acidic pH (De, Chattopadhyay, and Singh 2019; Prieto, Pineda, and Aguilar 1999). As depicted in Figure 1D, the total antioxidant capacity of all hydrolysates increased markedly by progressing the hydrolysis process (*p <* 0.05). Generally, the total antioxidant capacity of samples was in the range of 0.301–0.741 (absorbance at 695 nm). At the end of hydrolysis, the total antioxidant capacity of samples was in the order of GHs produced by pancreatin (0.741, Abs at 695 nm) > alcalase (0.521, Abs at 695 nm) = trypsin (0.51, Abs at 695 nm). These results showed that GH produced by pancreatin had the most favorable electron‐donating potential compared to other hydrolysates, leading to considerable capacity in radical chain termination and lipid oxidation prevention. It can be concluded that the degree of hydrolysis, hydrolysis time, and the applied enzyme undeniably affect the total antioxidant capacity of hydrolysates. Similar findings were reported in enzymatic hydrolysis of fenugreek, Turkmen melon seed, orange seed, smoothhound muscle, and wastes of skipjack fish processing (Alvand et al. 2022; Bougatef et al. 2009; Kaveh et al. 2022, 2023; Mazloomi, Mahoonak, and Houshmand 2019). Also, the positive effect of enzymatic hydrolysis on the total antioxidant capacity of protein hydrolysate was stated by Umayaparvathi, Arumugam, et al. (2014) and Aguilar et al. (2019) in the enzymolysis of oyster and black bean proteins, respectively (Aguilar et al. 2019; Umayaparvathi, Arumugam, et al. 2014).

### 3.3 Encapsulation of GH by Spray‐Drying

#### 3.3.1 Encapsulation Efficiency (EE)

**FIGURE 2:**
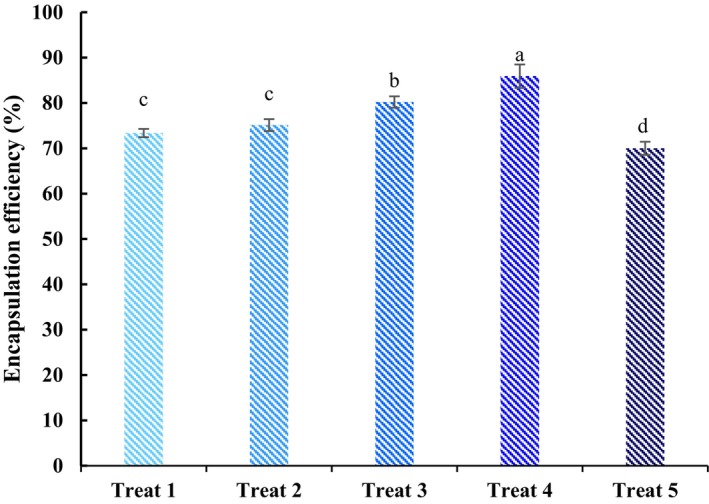
Encapsulation efficiency of spray‐dried microcapsules of gluten hydrolysate. *Different letters show significant difference at 5% level.

The EE of microcapsules is one of the most critical factors in determining the nutrient delivery system (Liu et al. 2020). This factor is related to the retention of the compound, which depends on the spray drying process conditions and the feed properties (Anandharamakrishnan 2015). As shown in Figure 2, 69.99%–85.79% EE was obtained depending on the wall materials used. According to our results, it was found that the increment of PS ratio in the composition of the wall material until the ratio of 30:70 MD/PS led to higher encapsulation efficiency. The spray‐dried microcapsules containing MD:PS at a ratio of 30:70 (treat 4) as wall material showed the highest EE (85.79%) (*p <* 0.05). Conversely, the lowest EE (96.99%) was obtained using MD:PS at a ratio of 0:100 (treat 5). The reduction in EE can be due to the aggregation of particles to each other and adhering to the spray dryer chamber wall (Hesarinejad et al. 2021). Generally, it has been documented that spray‐dried microcapsules' encapsulation efficiency depends on spray drying conditions, especially inlet air temperature, concentration and type of wall materials, and the ratio of wall‐to‐core (Kaul et al. 2022). In this study, the different EE may be associated with the different polymer matrices formed by the used wall materials that considerably affect the retention of the loaded compound and crust‐forming capacity (Etzbach et al. 2020). The high EE achieved by increasing PS in the wall materials of microcapsules can be due to the excellent film‐forming capability of starches, which positively affects the process of spray drying by avoiding the migration of loaded compound (core) to the particle's surface (Tonon et al. 2012). The findings were similar to the results of Carneiro et al. (2013), Kaul et al. (2022), and Tonon et al. (2012) in microencapsulation of flaxseed oil, iron and zinc, and flax seed oil, respectively. Also, Esquivel‐González et al. (2022) reported that increasing the proportion of sweet potato starch in the wall materials resulted in the increase of EE of spray‐dried betanin. They attributed this result to the reduction of particle stickiness, preventing adhering to the spray dryer chamber wall (Esquivel‐González et al. 2022).

#### 3.3.2 Moisture Content and Water Activity (Aw)

**FIGURE 3:**
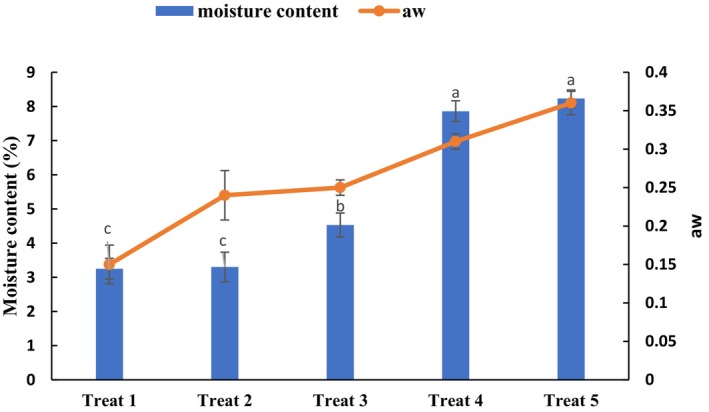
Moisture content and aw of spray‐dried microcapsules of gluten hydrolysate.

Aw and moisture content are important factors in spray‐dried microcapsules as they directly affect their storage stability. It has been documented that powders with a moisture content of ≤ 5% are favorable (Premi and Sharma 2017). On the other hand, aw is related to the free water of the product and thus is an essential parameter in the shelf‐life stability of the powders. Desirable amounts of aw can control the spoilage by microorganisms and lipid oxidation (Júnior et al. 2023). High aw leads to lower stability and higher deterioration. Generally, aw ≤ 0.6 is recommended for powders (Tuyen, Nguyen, and Roach 2010). The desired aw and moisture content level ensures spray‐dried microcapsules' microbiological and chemical stability (da Silva et al. 2013). Figure 3 shows the moisture content and aw of GH microcapsules. Microcapsules' aw and moisture content ranged from 0.14% to 0.36% and 3.8%–8.2%, respectively. Generally, as PS concentration increased in the feed solution, the aw and moisture content increased significantly (*p* < 0.0.5). This may be attributed to the higher concentration of MD than PS, as reducing the ratio of MD in the feed solution reduces feed solids, resulting in lower evaporation capacity and higher moisture content of powders (Mishra, Mishra, and Mahanta 2014). In general, all samples except treat 4 and treat 5 had moisture content below 5% and aw below 0.6; these values confirm the high microbiological stability of the GH microcapsules. These results were in the range of findings reported by Shadordizadeh, Mahdian, and Hesarinejad (2023). They revealed that the moisture content and aw of freeze‐dried powder of *Indigofera tinctoria* extract were 1.21–3.14 and 0.182–0.241, respectively (Shadordizadeh, Mahdian, and Hesarinejad 2023). Moreover, these findings are in accordance with Esquivel‐González et al. (2022), Tonon et al. (2012), and Fazaeli et al. (2012) in microencapsulation of betanin, flax oil, and black mulberry juice, respectively.

#### 3.3.3 Solubility

Favorable solubility is a critical factor for the use of powders in the food's formulation. So, solubility is an important quality factor of spray‐dried powders, which shows the particle's behavior in the water phase and determines the reconstitution capability (Fernandes et al. 2008). The GH microcapsules showed an excellent solubility of at least 85% (Table 1), but there were considerable differences between samples (*p* < 0.05). Generally, a higher proportion of MD than PS in the carrier composition resulted in higher solubility, as the lowest solubility (85.43%) was related to treat 5, which contained the lowest amount of MD among other treatments. Treats 1 (96.68%) and 2 (95.71%) had the highest solubility with no significant differences (*p* > 0.05). The favorable solubility of treatments containing a high proportion of MD can be related to the hydrophilic nature of MD. Although treatments containing a high proportion of MD had higher solubility, treatments containing higher amounts of PS also had considerable solubility (> 85%). It can be related to the PS's amphiphilic character helped the dispersion and the interaction with non‐polar and polar components (da Silva et al. 2013). Similar to these results, Caliskan and Nur Dirim (2013), Nadeem, Torun, and Özdemir (2011), and Kaul et al. (2022) reported that higher concentration of MD in the carrier composition led to a higher solubility of spray‐dried sumac extract, mountain tea water extract, and iron and zinc nano capsules. Also, Don et al. (2024) reported that the use of maltodextrin or modified starch as wall material led to favorable solubility of spray‐dried black pepper oleoresin (Don et al. 2024). Contradict to these results, Kaveh et al. (2018) revealed that increasing the maltodextrin concentration in the carrier, the solubility of spray‐dried powder reduced (Kaveh et al. 2018). These different results can be related to the differences in the nature of spray dried compound and the process condition. In this regard, it is documented that the solubility of spray‐dried powders depends on different factors, including the physicochemical properties of the loaded compound, the interaction between encapsulated compounds and wall materials, concentration and type of carriers, drying conditions, and the temperature of inlet air (Kaveh et al. 2018; Wardhani et al. 2020).

**TABLE 1:** The solubility, hygroscopicity, bulk, and tapped density of GH microcapsules.

	Treat 1	Treat 2	Treat 3	Treat 4	Treat 5
Solubility (%)	96.68 ± 1.18^a^	95.71 ± 0.55^a^	91.43 ± 1.06^b^	89.20 ± 0.66^b^	85.43 ± 1.17^c^
Hygroscopicity (%)	17.80 ± 1.18^d^	18.82 ± 0.89^d^	20.45 ± 0.36^c^	22.78 ± 0.36^b^	26.65 ± 1.14^a^
Bulk density (g/ml)	0.49 ± 0.03^a^	0.48 ± 0.02^a^	0.40 ± 0.01^b^	0.37 ± 0.02^bc^	0.36 ± 0.01^c^
Tapped density (g/ml)	0.65 ± 0.02^a^	0.60 ± 0.04^a^	0.54 ± 0.03^b^	0.49 ± 0.01^c^	0.44 ± 0.02^d^

*Note:* Different letters in each row show significant differences at 5% level.

#### 3.3.4 Hygroscopicity

Hygroscopicity is a qualitative measurement of the way in which powders absorb moisture from the environment, which determines their ease of handling and storage stability (Kaul et al. 2022). Absorbing moisture leads to the formation of liquid bridges between spray‐dried particles, resulting in chemical and microbial reactions and, consequently, loss of functional properties of the powders (Kaveh et al. 2018). Table 1 presents the hygroscopicity values of GH microcapsules in the 17.80%–26.65% range. Higher concentrations of MD reduced the hygroscopicity values of microcapsules as the highest and lowest hygroscopicity values were related to treats 5 and 1, which contain 100% MD and PS, respectively (*p <* 0.05). This may be due to the low water absorption capacity of MD and the high hygroscopic nature of PS (Wang et al. 2020). It has been stated that the hygroscopicity values of powders depend on the product's inherent compositions, the carriers' type and concentrations, and drying conditions (Kaveh et al. 2018). It has been documented that the powder's desirable hygroscopicity value is below 20%. So, the hygroscopicity values for the GH microcapsules of treats 1 and 2 were in the desirable range, and the value of treat 3 was in the critical limit. It can be concluded that the combination of PS with MD in a proportion volume ratio less than 50:50 led to the production of microcapsules with favorable storage stability and hygroscopic properties. Also, Kaveh et al. (2018) reported that increasing MD concentration from 10% to 30% led to a reduction in hygroscopicity of spray‐dried stevia powder (Kaveh et al. 2018). The same tendency was observed by Esquivel‐González et al. (2022) and Rodríguez‐Díaz, Tonon, and Hubinger (2014) in spray drying of betanins and protein hydrolysate of blue shark skin. They reported higher concentrations of MD led to lower hygroscopicity values of spray‐dried microcapsules. The reduction effect of MD may have resulted from its film‐forming capacity with low hygroscopicity and the increasing effect on the total glass transition temperature (Fang and Bhandari 2012).

#### 3.3.5 Density (Tapped and Bulk and Density)

Tapped and bulk density are critical factors from economic and transportation points of view. These factors are related to the powders' storage, packaging, and flowability (Kaveh et al. 2018). GH microcapsules tapped and bulk density ranged between 0.44–0.65 (g/ml) and 0.36–0.49 (g/ml). Generally, treats 4 and 5 with lower solid content had lower densities than treats 1 and 2 with higher solid content (*p <* 0.05). In this regard, it has been stated that an increase in feed concentration results in an increase in the particle size and, consequently, a decrease in the powder's density (Goula and Adamopoulos 2004). Also, the higher density of powders produced by higher concentrations of MD as carriers can be attributed to the particle structural collapse and high degree of agglomeration, which decrease the volume of the powders and consequently increase their density (Bae and Lee 2008). Also, it is stated that by increasing the concentration of carriers, the viscosity of inlet feed increases, leading to increasing the particle size and decreasing the density of particles (Kaveh et al. 2018). Similar findings were revealed by Rodríguez‐Díaz, Tonon, and Hubinger (2014) in the encapsulation of blue shark skin protein hydrolysate, but Kurozawa, Park, and Hubinger (2011) reported lower density values by increasing MD in the encapsulation of chicken meat protein hydrolysate. These contradictory results can be related to the differences in the wall material's composition, drying conditions, and inlet air temperature.

#### 3.3.6 Release Profile During SGF and SIF

**FIGURE 4:**
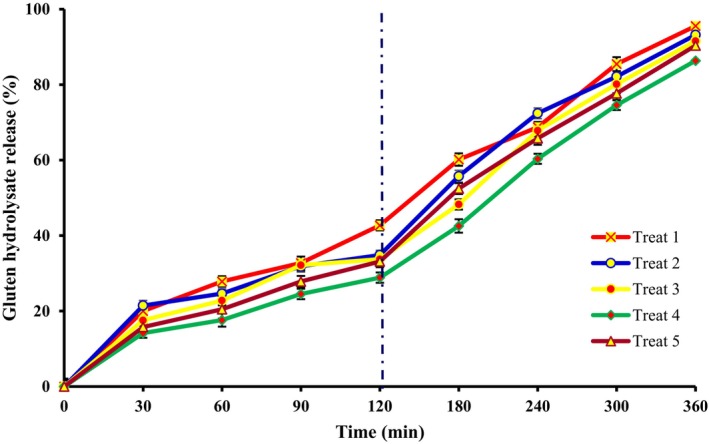
Release profile of gluten hydrolysate under simulated gastrointestinal digestion conditions.

Evaluation of the release behavior of the active compounds is essential to determine their bioavailability and stability (Shamaei et al. 2017). The evaluation of the release behavior of spray‐dried microcapsules during SGF and SIF is important to study their potential to be used for the targeted delivery of loaded compounds (Chew et al. 2015). The use of in vitro digestion—a model for evaluating the delivery of loaded bioactive compounds is a suitable alternative to in vivo methods because it is less time‐consuming and has no ethical issues (Calvo et al. 2012). The release behavior of GH microcapsule under SGF and SIF is shown in Figure 4. The release amount increased significantly during gastric and intestine digestion (*p* < 0.05). At the end of the SGF digestion, the highest (42.71%) and lowest (28.86%) releases were related to treat 1 and treat 5, respectively. At the first 1 h of SIF digestion, the release behavior was in the order of treat 1 > treat 2≈, treat 5 > treat 3 > treat 4. Generally, the use of MD or PS alone as wall materials did not have a desirable protective effect, but their combination, especially at the ratio of 30:70 (treat 4), significantly retard the release of GH. The high release rate of GH observed in treat 1 can be related to the structure of MD that degraded more quickly than PS by α‐amylase at the initial stage of digestion. Moreover, as mentioned, treat 1 had the highest solubility due to the presence of MD. This high solubility increases the contact of microcapsules with water, which can destroy their structure and, consequently, the high release of active compounds. So, the combination of MD and PS led to the formation of a more complex and stable structure, which could considerably protect the GH from enzymatic digestion (Norkaew et al. 2019). Similar findings were reported by Ding et al. (2019), Bao et al. (2023), and Norkaew et al. (2019) in the spray drying of soybean oil, essential oil, and black rice anthocyanin, respectively. Also, He et al. (2016) revealed that the use of octenyl‐succinic anhydride starch and xanthan gum as wall material at a ratio of 60:1 (*w*/*w*) in spray drying of conjugated linoleic acid could effectively control the release rate at SGF and SIF (He et al. 2016).

#### 3.3.7 Surface Morphology Analysis by SEM

**FIGURE 5:**
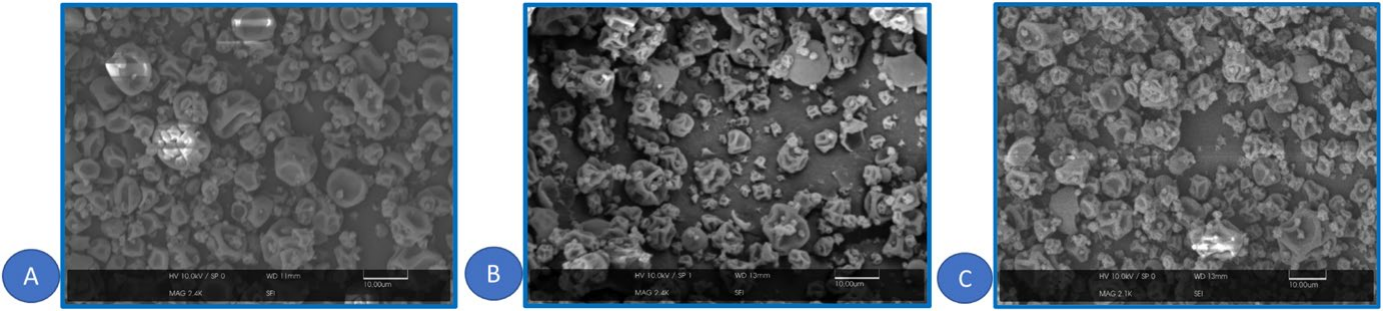
The SEM images of GH microcapsules (A) treat 1, (B) treat 4, and (C) treat 5.

As shown in Figure 5, the microcapsules had approximately the same morphology with a spherical shape and wrinkled surface. In this regard, it has been documented that the mechanical stresses due to uneven drying at different parts of the liquid droplets during the first drying step can produce wrinkled particles (Klinkesorn et al. 2006). Visible surface indentations result from using polysaccharides as wall materials, usually reported whenever they are used as wall material (Kaul et al. 2022). Similarly, He et al. (2016) stated no differences between the morphology of conjugated linoleic acid microcapsules produced by different wall materials. Moreover, spray‐dried microcapsules with the same shape were revealed by Li et al. (2020) and Catalkaya, Guldiken, and Capanoglu (2022) in the encapsulation of volatile ethyl acetate and black chokeberry. Similar to these findings, Don et al. (2024) revealed that spray‐dried black pepper oleoresin samples were rather spherical in shape without cracks or fractures (Don et al. 2024). Also, Norkaew et al. (2019) reported that spray‐dried black rice anthocyanin had a similar shrink shape with different degrees of concavity. They attributed the formation of concavities on the microcapsule's surfaces to uneven shrinkage forces due to the rapid evaporation by inlet hot air (Norkaew et al. 2019).

## 4 Antioxidant Stability of Microcapsules During Storage at 25°C

**FIGURE 6:**
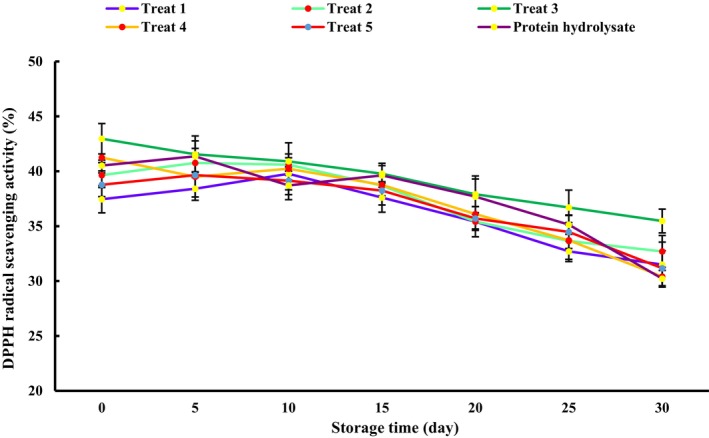
DPPH radical scavenging activity of gluten microcapsules during storage at 25°C.

From the industrial point of view, the stability of bioactive compounds is essential during their food and pharmaceutical application, ensuring a constant level of product quality (Zier, Schultze, and Leopold 2018). The DPPH radical scavenging ability of all microcapsules and free GH hydrolysate was decreased markedly during 30 days of storage (*p* < 0.05) (Figure 6). The decrease in the antioxidant potential of free GH was considerably higher than that of GH microcapsules (*p* < 0.05). These results showed the protective effect of encapsulation by spray drying with MD and PS. On the 30th day of storage, the highest antioxidant capacity (35.47%) was related to Treat 3, and Treat 4 showed the lowest antioxidant activity (30.41%). This shows the high protective effect of MD and PS at a ratio of 50:50 than their use alone or at a ratio of 30:70. Moreover, it can be a sign of higher entrapping efficiency of GH in treat 4 compared to the other samples. Antioxidant stability of spray‐dried flax seed protein hydrolysates and soy peptide fractions were reported by Sarabandi and Jafari (2020) and Akbarbaglu et al. (2023), respectively (Akbarbaglu et al. 2023; Sarabandi and Jafari 2020). Similar results were reported by Cassol and Noreña (2021) and Hamin Neto et al. (2014) in encapsulation of bioactive compounds of *Hibiscus sabdariffa* and *Eupenicillium javanicum* peptidases, respectively. Also, Bhattacherjee, Tandon, and Dikshit (2014) reported that the loss of Fe reducing power of spray‐dried aonla juice (41.9%) was significantly lower than the aonla juice (56.9%) at room temperature during 12 months (Bhattacherjee, Tandon, and Dikshit 2014).

## 5 Conclusion

In recent years, the tendency for functional foods with the least synthetic additives has increased significantly. In this regard, protein hydrolysates are ideal options that have various health‐beneficial activities. However, their application is limited due to their sensitivity to undesirable conditions and uncontrolled delivery. Spray drying is a suitable technique to face these challenges. The results of this study showed that GH had significant antioxidant potential and GH spray drying using MD and PS had positive effects on physicochemical properties and release behavior during SGF and SIF. Using MD and PS at a 30:70 ratio resulted in a high EE (85.89%) with desirable physicochemical properties, including moisture content, solubility, density, and hygroscopicity. Based on SEM images the GH microparticles had the same morphology with a spherical shape and wrinkled surface. The use of MD and PS at a 30:70 ratio increased the stability of GH during storage time and controlled its release during digestion. Generally, the results of this research indicated the efficiency of enzymolysis in the production of antioxidant peptides and the importance of the spray‐drying technique by use of MD and PS as cheap polysaccharides in producing microcapsules with desirable stability, controlled release profile, and physicochemical properties. So, the spray‐dried GH microcapsules with suitable stability and antioxidant activity can be used in food product formulations as natural antioxidants. Moreover, the spray‐dried microparticles have a high potential for application in pharmaceutical and cosmetic fields.

## Author Contributions

**Benyamin GanjiVtan:** methodology (equal), writing – original draft (equal). **Seyyed Hossein Hosseini Ghaboos:** conceptualization (equal), writing – review and editing (equal). **Alireza Sadeghi Mahoonak:** conceptualization (equal). **Taher Shahi:** visualization (equal). **Neda Farzin:** conceptualization (equal).

## Conflicts of Interest

The authors declare no conflicts of interest.

## Data Availability

All data generated or analyzed in this article are included within it.

## Endnotes

^1^
1,1‐diphenyl‐2‐picrylhydrazyl.
